# Short-term outcomes of ulnar, median, tibial, and common fibular neurolysis in leprosy neuropathy: A prospective study of pain, sensory function, and muscle strength

**DOI:** 10.1371/journal.pntd.0014556

**Published:** 2026-07-23

**Authors:** Thiago Montenegro da Silva, Jaqueline da Silva Mendes, Amanda Gabrielle dos Santos Cordeiro, Raquel da Mata Serique, Alexandra de Freitas Costa, Carolina Talhari, Luiz Carlos de Lima Ferreira, Hélio Amante Miot, Marcelo Ribeiro Alves, Flavio Alves Lara, Sinésio Talhari

**Affiliations:** 1 Programa de Pós-Graduação em Ciências Aplicadas à Dermatologia, Universidade do Estado do Amazonas Manaus, Manaus, Brazil; 2 Fundação Hospitalar Alfredo da Matta, Manaus, Brazil; 3 Universidade Estadual Paulista, FMB-UNESP, Botucatu, Brazil; 4 Instituto Nacional de Infectologia (IPEC), Fundação Oswaldo Cruz, Rio de Janeiro, Brazil; 5 Lab. de Microbiologia Celular, Instituto Oswaldo Cruz, Fundação Oswaldo Cruz, Rio de Janeiro, Brazil; University of Bremen: Universitat Bremen, GERMANY

## Abstract

**Background:**

Leprosy neuropathy is a major cause of persistent pain and functional impairment. When neuritis does not respond adequately to medical management, open neurolysis is often used to decompress inflamed peripheral nerves, yet short-term outcomes across different nerve groups remain incompletely characterized.

**Methodology/Principal findings:**

We conducted a prospective cohort study including 65 patients with clinically diagnosed leprosy neuropathy who underwent open neurolysis of the ulnar, median, tibial, or common fibular nerves. Sensory function, motor strength, and neuropathic pain were evaluated preoperatively and at 30 and 60 days postoperatively using Semmes–Weinstein monofilaments, the Medical Research Council scale, and a visual analog scale, respectively. Changes over time were estimated using mixed-effects cumulative link models for ordinal outcomes, adjusted for age, operated side, and nerve group, with patient ID as a random effect. The contralateral limb was assessed at the same time points as a within-patient comparator. Pain decreased significantly at the group level in all operated nerve groups at 30 days (p < 0.001), and this reduction was maintained at 60 days under routine postoperative care, although individual responses varied. Sensory improvement was observed only after common fibular neurolysis at 30 days and was not sustained at 60 days. No measurable motor improvement was detected during this early 60-day follow-up period. Pain reduction did not differ significantly between patients operated more than three years after completion of multidrug therapy and those operated earlier, although pain worsening was observed only in the < 3-year group.

**Conclusions/Significance:**

Overall, these findings support neurolysis primarily as a short-term analgesic procedure within the early postoperative period, while the absence of measurable motor improvement should be interpreted as a preliminary short-term observation. Longer-term controlled studies are needed to refine surgical indications and determine whether delayed sensory or motor recovery occurs after neurolysis.

## Author summary

Leprosy (Hansen disease) can damage peripheral nerves and leave people with lasting pain, loss of sensation, weakness, and disability of the hands and feet. When nerve inflammation (neuritis) does not respond well to medical treatment, surgeons may decompress the affected nerve (neurolysis) in an attempt to relieve symptoms and prevent further damage. However, it remains uncertain how much neurolysis improves pain and function in different nerve groups, particularly in the short term. In this prospective study, we followed 65 patients with leprosy neuropathy who underwent open neurolysis of the ulnar, median, tibial, or common fibular nerves. We assessed pain, sensation, and muscle strength before surgery and again at 30 and 60 days. At the group level, pain decreased significantly after surgery across all operated nerve groups, and this reduction was maintained over the 60-day follow-up period, although individual responses varied. In contrast, sensory improvement was limited and transient, and we did not observe measurable gains in muscle strength within the same timeframe. Taken together, these findings suggest that, in leprosy neuropathy, neurolysis should be understood primarily as a short-term procedure for pain relief rather than one that reliably restores lost sensory or motor function. This distinction can help guide surgical decision-making and set realistic expectations during patient counselling.

## Introduction

Leprosy is one of the oldest and most stigmatized chronic diseases. It is caused by two mycobacteria: *Mycobacterium leprae* and *Mycobacterium lepromatosis*. Clinically, it is characterized by localized or disseminated skin lesions and frequently involves peripheral nerves. Without proper treatment, peripheral nerve damage can lead to loss of sensation in skin lesions, motor dysfunction, claw hands, palmar and plantar ulcers, muscle atrophy, and consequent difficulties in walking and impaired manual or occupational abilities [1,2].

In 2024, the World Health Organization (WHO) reported 172,717 new leprosy cases worldwide. Of these, 9,124 were newly diagnosed with grade 2 disability (G2D), a marker of delayed case detection and established visible impairments of the eyes, hands, and/or feet. Such disability largely reflects nerve damage that is often not fully reversible. Importantly, WHO emphasizes that nerve function impairment can still deteriorate during and after treatment: among cases in which nerve function assessment was recorded at treatment completion, 923/27,487 (3.4%) showed worsening, underscoring the need for close monitoring and timely interventions during and beyond multidrug therapy [2].

This global burden is especially relevant to Brazil. In the Brazilian Ministry of Health’s most recent consolidated report (2015–2024 series), Brazil recorded 22,129 new cases in 2024, corresponding to a detection rate of 10.41 per 100,000 inhabitants (classified nationally as a “high” level). Moreover, 11.5% of new Brazilian cases already had grade 2 physical disability at diagnosis, reinforcing how frequently patients still reach care after substantial neural damage has occurred [3].

Peripheral nerve injury in leprosy is multifactorial. It reflects both the neurotropism of *M. leprae*, including PGL-I–mediated interactions with Schwann cells, and the host inflammatory response that accompanies neuritis and reactional episodes. As the affected nerve becomes inflamed and enlarged, edema within anatomically constrained sites may increase endoneurial pressure, compromise microvascular perfusion, and contribute over time to ischemia, fibrosis, and axonal loss [4–6]. In this setting, neurolysis has been used as an adjunctive procedure for patients with persistent or progressive neuropathic pain or nerve-function impairment despite adequate medical treatment. By releasing constrictive perineural structures, the procedure aims primarily to relieve mechanical compression and improve the local environment of the nerve; whether this translates into sensory or motor recovery depends largely on the duration and severity of the underlying nerve damag [7].

Neurolysis is a surgical procedure designed to release a compressed nerve [8,9]. One consequence of the inflammatory process in leprosy is the infiltration of inflammatory cells into the endoneurium, perineurium, and epineurium, leading to compression of both myelinated and unmyelinated nerve fibers [5]. This progresses to fibrosis, further contributing to neural damage [9]. The compression also causes ischemic nerve injury, aggravating neuropathy [10,11].

External nerve compression occurs primarily due to nerve thickening, which may be intermittent, partial, or complete. The thickening can develop uniformly along the nerve’s peripheral pathway or appear fusiform or beaded (resembling a rosary). In tuberculoid nerve granulomas, caseous necrosis may occur, resulting in complete nerve destruction due to abscess formation. These abscesses contribute to the compression of nerve fascicles [12].

Studies show that when performed at the appropriate time, preferably before irreversible axonal damage occurs, the technique can contribute to pain reduction, recovery of sensitivity, and muscle strength [7,9,10,13,14]. However, its outcomes vary depending on the affected nerve, duration of compression, and technique used.

This prospective study estimated short-term changes in sensory function, muscle strength, and neuropathic pain after neurolysis of the ulnar, median, tibial, and common fibular nerves in patients with leprosy neuropathy. Because these outcomes are ordinal and were measured repeatedly over time, we used mixed-effects cumulative link models to account for within-patient correlation and to estimate postoperative changes at 30 and 60 days. By examining different nerve groups separately, we sought to clarify which clinical outcomes are most likely to improve in the early postoperative period and to provide evidence that may support surgical decision-making and more realistic preoperative counselling.

Understanding which patients will benefit from this invasive procedure is crucial for guiding clinical and surgical decisions, aiming to minimize the disabling sequelae of this neglected disease more effectively.

## Methods

### Ethics statement

This study was approved by the Alfredo da Mata Hospital Foundation Ethics Committee (CAAE: 85379624.9.0000.0002), and all participants provided written informed consent.

### Study design

This is a prospective study conducted at the Alfredo da Mata Hospital Foundation (FUHAM) enrolling patients aged 18 or older, of any sex, diagnosed with leprosy neuropathy involving the ulnar, median, tibial, and/or common fibular nerves, who underwent conventional open decompression surgery (*in situ* release) at FUHAM between January 2020 and December 2024. We included patients with leprosy-related nerve involvement who underwent surgical intervention after lack of response to clinical treatment, defined as persistence or progression of neurological symptoms, including pain, sensory loss, or motor deficit, despite adequate drug management with corticosteroids and/or other therapies according to the institutional protocols of FUHAM.

Alternative etiologies of neuropathy were assessed at baseline through clinical evaluation and review of medical records. The diagnosis of leprosy neuropathy was supported by the documented diagnosis and clinical classification of leprosy, standardized sensory-motor mapping, nerve thickening and/or pain on nerve palpation, the anatomical distribution of sensory or motor impairment, and persistence or progression of neurological symptoms despite clinical management. Most patients had leprosy forms with cutaneous involvement documented at diagnosis, whereas patients with pure neural leprosy were included only when they showed a focal or asymmetric pattern of nerve involvement, with nerve thickening and/or pain on palpation, in a leprosy-endemic setting. Medical records were reviewed for other potential causes of neuropathy, including diabetes, alcohol-related neuropathy, orthopedic compressive syndromes, radiculopathies, previous local surgical procedures, and clinical features suggesting systemic or inflammatory neuropathic disorders. Patients were not included when the neuropathy was better explained by another etiology. Diabetes itself was not considered an automatic exclusion criterion; rather, patients with diabetes were retained only when the neuropathy pattern was focal or asymmetric and clinically consistent with leprosy neuropathy rather than length-dependent diabetic polyneuropathy. When available, electromyography reports were reviewed to support the clinical diagnosis and to exclude alternative primary neuropathic etiologies.

Reoperations were not included to avoid structural bias.

### Patient evaluations procedures

A series of evaluations was carried out using the Sensory-Motor Mapping form, developed to assess patients undergoing nerve decompression surgery (neurolysis) at FUHAM, Manaus, Amazonas state, Brazil.

These evaluations were performed by trained physiotherapists experience leprosy neuropathy following the Brazilian Ministry of Health simplified neurological assessment protocol [6]. They included standardized assessments of sensory perception and motor strength in the hands and feet, as well as pain intensity using the Visual Analog Scale (VAS) [15]. All procedures were explained to the participants before testing.

The contralateral limb was examined in parallel with the operated limb at the same time points (preoperatively, and at 30 and 60 days) and using the same standardized instruments for sensory testing, motor strength grading, and pain assessment. We used these paired measurements as a within-patient comparator, because they provide a practical reference under the same systemic disease context, rehabilitation schedule, and follow-up conditions. Importantly, given that leprosy neuropathy may be bilateral or clinically silent in early stages, contralateral limbs were not assumed to be disease-free; rather, they were treated as a comparator assessed with identical methodology, allowing postoperative changes in the operated limb to be interpreted in relation to contemporaneous measurements from the opposite side.

Assessments were performed at three distinct time points: preoperatively (before the surgical procedure), and at 30 and 60 days post-surgery. The study form captured sex, age, time elapsed between leprosy diagnosis and surgery, and the clinical form of leprosy at diagnosis, as recorded in the Brazilian Ministry of Health SINAN (Notifiable Diseases Information System) leprosy notification/investigation form: indeterminate, tuberculoid, borderline, lepromatous, and pure neural leprosy [1,16].

For sensory evaluation, esthesiometry was conducted using Semmes-Weinstein monofilaments (SW) [6]. The test began with the thinnest filament (0.05 g/cm^2^, green). If no response was detected, progressively thicker monofilaments were used in the following sequence: 0.2 g/cm^2^ (blue), 2 g/cm^2^ (violet), 4 g/cm^2^ (dark red), 10 g/cm^2^ (orange), and finally 300 g/cm^2^ (magenta red). The nylon filament was applied perpendicularly to the skin surface, with pressure maintained for 1–2 seconds until the filament bent, without sliding across the skin.

The green (0.05 g/cm^2^) and blue (0.2 g/cm^2^) monofilaments were applied three times at each specific point, while the remaining filaments were applied once per point. Sensory responses were recorded using a scale of numbness: 1 for green (0.05 g/cm^2^), 2 for blue (0.2 g/cm^2^), 3 for violet (2 g/cm^2^), 4 for dark red (4 g/cm^2^), 5 for orange (10 g/cm^2^), 6 for magenta red (300 g/cm^2^), and 7 when the magenta red filament was not perceived.

Motor strength was assessed in muscles innervated by the ulnar nerve (abductor digiti minimi) and the common fibular nerve (tibialis anterior). Muscle strength was graded using the Medical Research Council (MRC) scale, ranging from 0 (no visible or palpable contraction) to 5 (normal strength): 1, flicker/trace contraction; 2, active movement with gravity eliminated; 3, active movement against gravity; and 4, active movement against gravity with resistance [6].

### Ulnar nerve decompression at the elbow

For the surgical procedure, the patient was placed in the supine position, with the upper limb supported on the surgical table and the elbow flexed at an angle of 120° to 130°. Local anesthesia was administered by infiltration with 2% lidocaine. An L-shaped skin incision was made on the medial aspect of the elbow, posterior to the medial epicondyle. The deeper tissue layers were dissected to access and proximally release the ulnar nerve between the septum and the anteromedial border of the triceps muscle. The Osborne ligament, which extends from the olecranon to the medial epicondyle and forms the roof of the cubital tunnel, was incised along its medial edge. Distal decompression proceeded until the muscular arch of the flexor carpi ulnaris was opened. The skin was closed with interrupted 3-0 nylon sutures.

### Median nerve decompression at the wrist

The patient was positioned supine, with the upper limb in supination and supported on the surgical table. Local anesthesia was administered by infiltration with 2% lidocaine. A longitudinal skin incision was made on the volar aspect of the wrist using a No. 15 scalpel blade, extending distally into the palm. After incising the deeper planes, care was taken to preserve veins and nerve branches in the area. The volar and transverse carpal ligaments were released using a No. 15 scalpel, following the ligament fibers distally up to the vascular arch in the palm. The skin was closed with interrupted 3-0 nylon sutures.

### Tibial nerve decompression

With the patient in the supine position and the lower limb extended, local anesthesia was administered via infiltration with 2% lidocaine. A longitudinal incision was made with a No. 15 scalpel blade on the medial aspect of the ankle, midway between the posterior region of the medial malleolus and the medial border of the Achilles tendon. The incision was extended through the deeper planes to reach the fibers of the flexor retinaculum (laciniate ligament). Dissection continued proximally above the medial malleolus and distally to the point where the tibial nerve branches into the medial and lateral plantar nerves. The skin was closed with interrupted 3-0 nylon sutures.

### Common fibular nerve decompression at the fibular neck

The patient was placed in the lateral decubitus position, and local anesthesia was administered by infiltration with 2% lidocaine. An “L” -shaped skin incision was made approximately 1 cm posterior to the head of the fibula. The deeper tissue layers were dissected to expose the common fibular nerve. Using a No. 15 scalpel blade, the fibrous arch at the origin of the peroneus longus muscle through which the common fibular nerve passes distally along the leg was released. The procedure was completed with skin closure using interrupted 3-0 nylon sutures.

### Postoperative protocols

In the postoperative period, the operated limb was immobilized according to the procedure performed. Patients returned to FUHAM at 7 days for the first dressing change and evaluation by the orthopedist. Sutures were removed, on average, at 21 days, when the physiotherapy program was intensified with emphasis on stretching, progressive strengthening, and sensory re-education. Analgesics were prescribed as needed, and systemic corticosteroids were used when clinically indicated for leprosy neuritis, in accordance with institutional protocols. Because medication use was documented as part of routine clinical care rather than a study-controlled intervention, individual-level details (dose, tapering schedule, and duration) were not systematically captured for the present analysis. Clinical follow-up included standardized reassessments at 30 and 60 days after surgery, with monitoring of pain intensity, sensory function, motor strength, and overall clinical course.

### Statistical analysis

Data are presented as median and interquartile range (IQR) or absolute numbers and percentages, as appropriate. To assess the impact of neurolysis on sensory function, motor strength, and pain, we employed mixed-effects cumulative link models [17]. This method is suitable for analyzing ordinal outcome data (numbness scores, muscle strength grades, pain scores) while accounting for repeated measures within individuals over time (baseline, 30 days, 60 days post-surgery). The models estimated the marginal probabilities of improvement in outcome scores over time, adjusting for fixed effects including postoperative time point, operated nerve type, age, and operated side.

Patient-specific variability (random effects) was incorporated using patient ID as a random intercept to account for within-subject correlation. The primary outcome was the odds ratio (OR) for improvement (decrease in numbness score or pain score, increase in muscle strength grade) at each follow-up time point compared to baseline. Separate models were fitted for each nerve group (ulnar, tibial, common fibular) for sensory and pain outcomes, and hand (ulnar/median innervated) and foot (tibial/common fibular innervated) strength. The contralateral limb served as a within-patient comparator, assessed using the same protocol at all time points. To compare pain reduction between patients who underwent neurolysis more than 3 years after treatment discharge versus those operated during or within 3 years post-treatment, mean marginal time-point effects were estimated using similar, mixed-effects cumulative link models adjusted for fixed effects (age, operated side), and including patient ID as a random effect.

Statistical analyses were conducted using R software (version 4.5, R Core Team). Multiple variable analyses *p*-values were calculated by using chi-squared tests for categorical nominal variables, and non-parametric Kruskal-Wallis tests for numerical continuous variables. The Mann-Whitney U test was conducted to evaluate the effectiveness of the surgical procedure in reducing common fibular nerve numbness and pain between patients with a cure discharge of ≥ 3 years and those with a cure discharge below 3 years. Graphical representations were generated using GraphPad Prism 7 (GraphPad Software, San Diego, CA, USA). A P-value < 0.05 was considered statistically significant for all comparisons. Significance levels are indicated in the figures as follows: **** P < 0.001, *** P < 0.005, ** P < 0.05, and ns (not significant).

## Results

The study cohort comprised 65 patients with a median age of 46 years (IQR = 15) and a predominance of men (63.1%, n = 41), particularly in the ulnar and median nerve subgroup (Table 1).

**Table 1:** Demographic, descriptive, and clinical characteristics of the study population.

		General Data	Tibial and common fibular nerve	Median Nerve	Tibial Nerve	Ulnar Nerve	Ulnar and Median Nerve
**Age**		46 (IQR* = 15)	41.5 (IQR = 17.75)	45.5 (IQR = 2.5)	41 (IQR = 11)	49 (IQR = 0)	47.5 (IQR = 18)
**Gender**	Female	24 (36.9%)	8 (36.4%)	1 (50%)	1 (50%)	1 (100%)	13 (34.2%)
	Male	41 (63.1%)	14 (63.6%)	1 (50%)	1 (50%)	0 (0%)	25 (65.8%)
**Clinical Form**	Pure Neural	12 (18.5%)	3 (13.6%)	1 (50%)	0 (0%)	0 (0%)	8 (21.1%)
	Tuberculoid	8 (12.3%)	2 (9.1%)	1 (50%)	0 (0%)	0 (0%)	5 (13.2%)
	Borderline	38 (58.5%)	15 (68.2%)	0 (0%)	2 ( 100%)	1 (100%)	20 (52.6%)
	Lepromatous	7 (10.8%)	2 (9.1%)	0 (0%)	0 (0%)	0 (0%)	5 (13.2%)
**Operated Side**	Right	26 (40%)	6 (27.3%)	1 (50%)	0 (0%)	0 (0%)	19 (50%)
	Left	39 (60%)	16 (72.7%)	1 (50%)	2 (100%)	1 (100%)	19 (50%)

*IQR means Interquartile Range.

Surgical distribution revealed that 22 patients (33.8%) underwent neurolysis of both common fibular and tibial nerves, while the majority (58.4%, n = 38) received intervention on median and ulnar nerves. Only 5 cases (7.7%) involved single-nerve decompression.

Borderline leprosy represented the predominant clinical form (58.5%), with particularly high prevalence among common fibular-tibial nerve cases (68.2%). Pure neural leprosy accounted for 18.5% of cases, showing increased frequency in ulnar-median nerve involvement (21.1%). Lepromatous presentations were uncommon (10.8%). Notably, common fibular and tibial nerve decompressions showed marked left-side predominance (72.7% of cases).

In our patient cohort, only 18 of 65 (27.7%) could be considered chronic cases, presenting with neuropathic symptoms or nerve impairment more than three years after completing multidrug therapy. All remaining 47 patients (72.3%) underwent neurolysis less than three years after treatment discharge.

Medication use was frequent and reflected routine clinical care for leprosy neuritis, reactional states, postoperative pain, and comorbidities. Drug intake was recorded in 42/65 patients (64.6%) before neurolysis, 34/65 (52.3%) during the perioperative period, and 49/65 (75.4%) after neurolysis. Prednisone was the most frequent medication, recorded in 27 (41.5%), 17 (26.2%), and 17 (26.2%) patients before, during, and after neurolysis, respectively. Analgesics or non-steroidal anti-inflammatory drugs were recorded in 7 (10.8%), 8 (12.3%), and 24 (36.9%) patients, and medications for neuropathic pain in 5 (7.7%), 5 (7.7%), and 11 (16.9%) patients, respectively. Analgesic and anti-inflammatory drugs included paracetamol, meloxicam, and ibuprofen, whereas neuropathic pain medications included gabapentin, pregabalin, and amitriptyline. Thalidomide was recorded in 3, 3, and 4 patients, mainly in the context of erythema nodosum leprosum or mixed reactional episodes, and multidrug therapy in 6, 8, and 6 patients before, during, and after neurolysis, respectively. These categories were not mutually exclusive.

Leprosy reactions documented before neurolysis were observed in 17/65 patients (26.2%). No reactions were recorded among patients with tuberculoid leprosy (0/8). Among borderline patients, 11/38 (28.9%) had reactions, including 10 type 1 reactions and one mixed type 1/type 2 reaction. Among patients with pure neural leprosy, 2/12 (16.7%) had reactions, including one type 1 and one type 2 reaction. Among lepromatous patients, 4/7 (57.1%) had reactions, including three type 1 reactions and one type 2 reaction. These reactions were recorded as pre-neurolysis events. Treatment consisted mainly of prednisone for type 1 reactions, whereas prednisone combined with thalidomide was recorded for type 2 or mixed reactional episodes. Reaction-related therapies overlapped with the concomitant medication categories described above.

**Fig 1:**
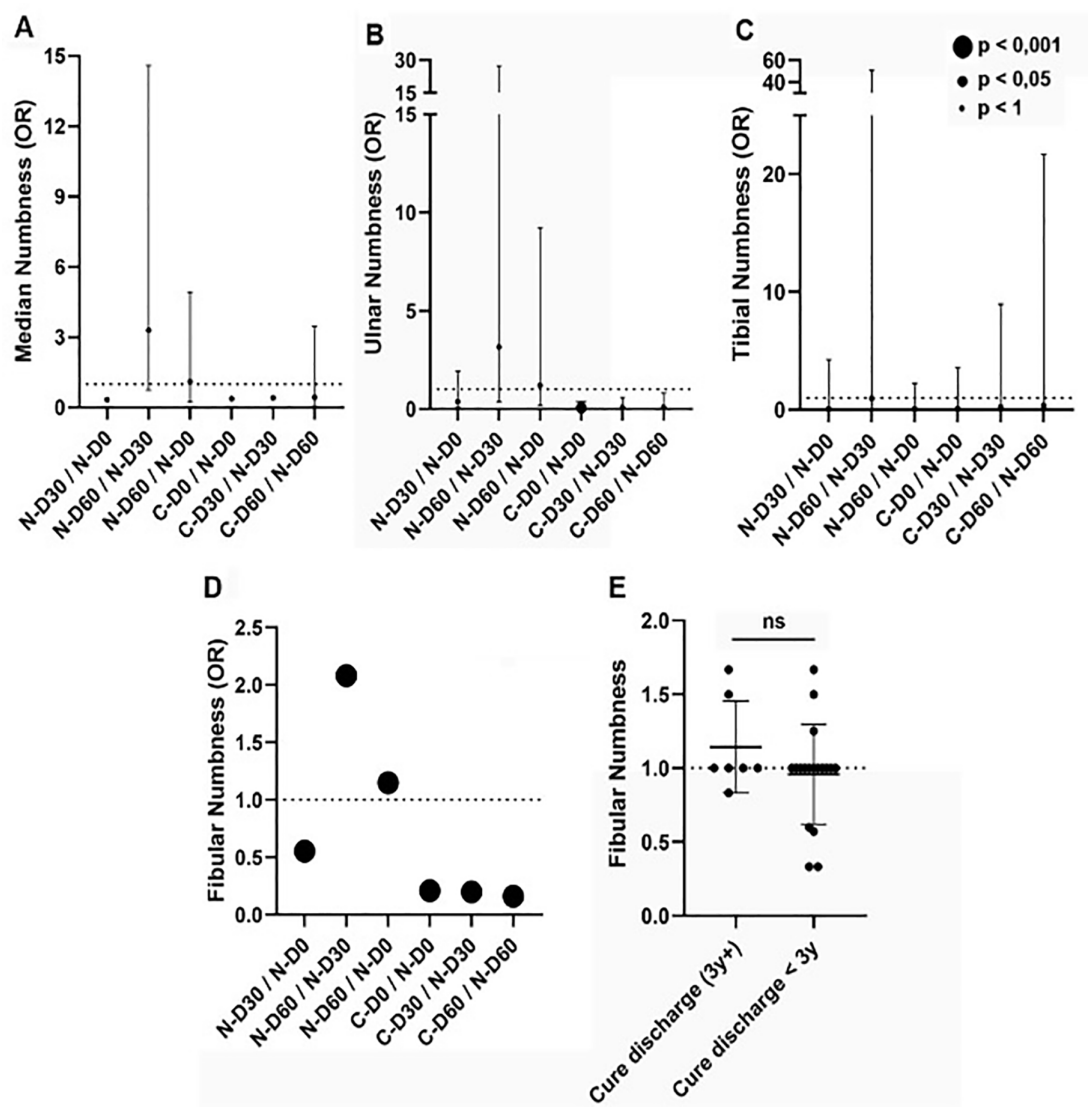
Follow-up assessing the impact of neurolysis surgery on numbness in leprosy patients. Numbness was evaluated in regions innervated by the median **(A)** ulnar **(B)**, tibial **(C)**, and common fibular **(D)** nerves before surgery (N-D0), 30 days post-surgery (N-D30), and 60 days post-surgery (N-D60) by the Semmes-Weinstein aesthesiometer. Contralateral limbs were assessed in parallel as within-patient comparators at corresponding time points (C-D0, C-D30, and C-D60). Mean marginal interaction effects over time were estimated using mixed-effects cumulative link models, adjusted for age and operating side (fixed effects). Patient identity was included as a random effect. Odds ratios (OR) are represented, with dashed lines indicating OR=1. Data was presented as median (interquartile range—IQR). Significance levels: large circle p < 0.001; medium circle p < 0.05. **E)** Effectiveness of the surgical procedure in reducing common fibular numbness performed in patients cured for 3 years or more, and in patients still undergoing treatment.

Following surgery, sensory function was assessed in the hands of patients who underwent neurolysis of the ulnar or median nerves, and in the feet of those who received intervention on the common fibular or tibial nerves. The odds ratio for changes in numbness, either reduction or increase, was calculated for the affected limbs (N) at 30 and 60 days postoperatively and compared with contemporaneous measurements from the contralateral limb (C), assessed using the same protocol at baseline, 30 days, and 60 days (Fig 1).

Notably (Fig 1D), only the common fibular nerve group demonstrated significant sensory improvement at 30-day follow-up (OR: 1.8, p < 0.001). However, this improvement proved transient, with the odds of improvement diminishing significantly when analyzed 60 days after intervention (OR: 0.87, p < 0.001). As anticipated, contralateral limbs consistently showed a lower degree of numbness compared to limbs affected by leprosy neuropathy.

**Fig 2:**
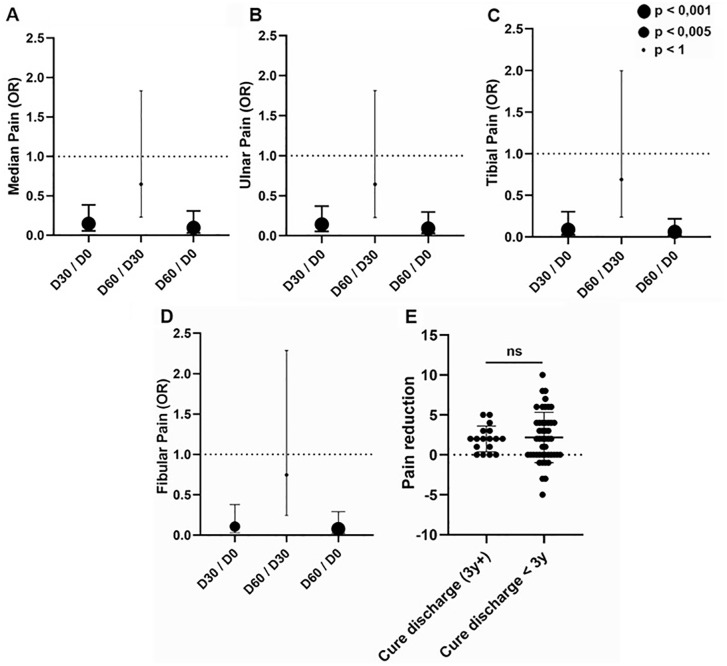
Follow-up assessing the impact of neurolysis surgery on pain in leprosy patients. Pain was evaluated in regions innervated by the median **(A)** ulnar **(B)**, tibial **(C)**, and common fibular **(D)** nerves before surgery (N-D0), 30 days post-surgery (N-D30), and 60 days post-surgery (N-D60) according to the self-reported pain numerical rating scale. Mean marginal time-point effects were estimated using mixed-effects cumulative link models, adjusted for age and operating side (fixed effects). Patient identity was included as a random effect. Odds ratios (OR) are represented, with dashed lines indicating OR=1. **E)** Pain reduction categorized into two patient groups: those who underwent neurolysis more than 3 years after treatment discharge (3y+), and those who received the procedure during treatment or before completing 3 years post-discharge (<3y). Significance levels: large circle p < 0.001; medium circle p < 0.005 and *ns* means not significant.

Subsequent analysis of pain outcomes revealed a significant reduction in neuropathic pain at the group level across all treated nerve groups at 30 and 60 days post-neurolysis (Fig 2). Thirty days after the procedure, we observed odds ratios for pain reduction of 6.77 (95% CI = 2.59; 17.72) in median nerves, 7.03 (95% CI = 2.71; 18.25,) in ulnar nerves, 11.36 (95% CI = 3.31; 39.01) in tibial nerves, and 9.42 (95% CI = 2.64; 33.62) in common fibular nerves.

Pain relief was maintained between the 30- and 60-day assessments. Compared with preoperative values, the odds of pain reduction at 60 days remained significantly higher across all operated nerve groups: median nerve, 10.45 (95% CI, 3.23–33.78); ulnar nerve, 10.95 (95% CI, 3.38–35.49); tibial nerve, 16.49 (95% CI, 4.59–59.29); and common fibular nerve, 12.62 (95% CI, 3.44–46.31). These findings indicate a sustained short-term analgesic effect of neurolysis in leprosy neuropathy.

To assess whether the timing of neuropathy influenced postoperative pain response, patients were stratified into two groups: those who underwent neurolysis more than three years after completion of multidrug therapy and those who underwent the procedure during treatment or within three years after treatment completion (Fig 2E). Pain reduction did not differ significantly between groups. However, 7 of 48 patients (14.6%) in the < 3-year group experienced worsening of pain, whereas this pattern was not observed among patients with longer-standing post-treatment neuropathy. In addition, 16 patients across both groups (24.2% of the cohort) did not experience pain reduction after surgery.

**Fig 3:**
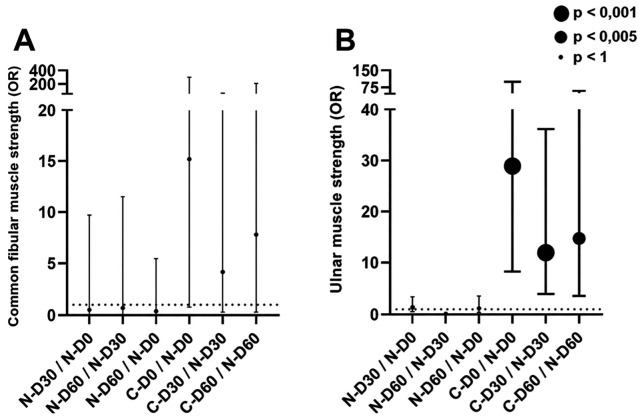
Short-term changes in muscle strength after neurolysis in patients with leprosy neuropathy. Muscle strength was assessed using the Medical Research Council scale before surgery (N-D0), 30 days after surgery (N-D30), and 60 days after surgery (N-D60). Foot strength **(A)** was evaluated in muscles innervated by the common fibular nerve, and hand strength **(B)** was evaluated in muscles innervated by the ulnar nerve. Contralateral limbs were assessed in parallel as within-patient comparators at the corresponding time points (C-D0, C-D30, and C-D60). Mean marginal time-point effects were estimated using mixed-effects cumulative link models adjusted for age and operated side, with patient ID included as a random effect. Odds ratios are shown, with dashed lines indicating OR = 1.

We then evaluated motor recovery in the operated extremities (Fig 3). Neurolysis was not associated with measurable gains in muscle strength within 60 days postoperatively. As expected, contralateral limbs maintained higher strength values throughout the study, reinforcing the limited short-term effect of neurolysis on motor recovery in this cohort.

## Discussion

Treatment of neuropathies in leprosy aims to control immunoinflammatory changes and prevent physical disabilities resulting from neural damage. Neurolysis is recommended for pain relief, improvement of neural function (motor and sensory), and prevention of deformities [1].

Neuropathic pain in leprosy has been reported across settings, with published estimates ranging from 17% to 70.3% [18]. In our cohort, men were more frequent (63.1%), consistent with the male predominance often observed in leprosy series.

Most patients were of working age, a pattern frequently observed in leprosy cohorts. In our sample, the median age was 46 years, reinforcing the social and occupational relevance of neuropathy-related disability in this population. In endemic settings, disability occurring during the productive years may have substantial consequences for patients and their families, highlighting the importance of timely strategies to prevent nerve damage and functional impairment [19].

Regarding clinical classification, 58.5% of patients in this study were borderline. Patients in the borderline spectrum are more likely to experience reactional episodes and neuritis, which are major drivers of acute nerve injury and disability [19–21]. Accordingly, they may be overrepresented among those who eventually require surgical decompression; however, the indication for neurolysis should be based on the presence of clinically significant nerve impairment and/or compression with persistent or progressive symptoms despite adequate medical management, rather than on clinical form alone [7,9,10,22].

Nerve thickening itself constitutes a major compressive factor, particularly where nerves pass through unyielding anatomical tunnels [12,23]. Our observations confirmed that even patients with mild peripheral neuropathy exhibited some degree of nerve thickening. Natural anatomical constriction points, such as the carpal tunnel at the wrist, Osborne’s ligament at the elbow, and tarsal tunnel at the ankle, frequently cause extrinsic compression due to nerve volume increase [12]. Surgical release of these compression points, as in any compressive syndrome, may critically influence functional prognosis [7,9,23].

The ulnar nerve is most frequently affected in leprosy [24], explaining the higher number of upper limb surgeries in our study. Our surgical technique avoided microneurolysis, preserving the epineurium and neural sheath, to maintain nerve vascularization and prevent soft tissue adhesion fibrosis. A study of 68 nerves found that avoiding fascicle exposure to adjacent soft tissues reduced adhesions and neural fibrosis [25].

Surgical decompression in leprosy neuropathy is primarily aimed at relieving entrapment of inflamed and enlarged nerve trunks at sites of anatomical narrowing, thereby reducing ischemic injury, alleviating neuropathic symptoms, and preventing further structural damage. External neurolysis is the most commonly performed procedure. It consists of decompressing the affected nerve at recognized entrapment sites, including the cubital tunnel for the ulnar nerve, the carpal tunnel for the median nerve, the tarsal tunnel for the tibial nerve, and the fibular tunnel or fascial structures around the fibular neck for the common fibular nerve. By releasing constrictive perineural tissues, external neurolysis may improve endoneurial blood flow, reduce ischemic injury, and restore nerve gliding within the surrounding soft tissues. In selected cases, intraneural abscesses or caseous granulomas may require drainage, sometimes combined with limited longitudinal epineurotomy, to reduce intraneural pressure. Although neurolysis has shown consistent benefit in alleviating neuropathic pain, sensory and motor recovery is less predictable and depends largely on the duration of nerve impairment and the extent of axonal loss. Optimal outcomes therefore require careful patient selection and timing, ideally before advanced fibrosis or irreversible axonal damage, and should be integrated with multidrug therapy, anti-inflammatory treatment for neuritis, corticosteroids when indicated, and rehabilitation. Together, these interventions form a multimodal strategy to reduce disability in patients with leprosy-related peripheral neuropathy [7,9,10].

Mixed-effects cumulative link models confirmed that common fibular neurolysis provided only transient sensitivity gains limited to the first postoperative month. This suggests temporary vascular decompression or acute neural adaptations may explain unsustained early improvement. In our cohort, no measurable gains in hand or foot muscle strength were detected within the 60-day follow-up. This finding should be interpreted as an early postoperative observation rather than evidence that neurolysis has no effect on motor recovery, because meaningful motor improvement after decompression may require a longer interval and depends on baseline axonal damage, remyelination, reinnervation, and rehabilitation. Thus, while neurolysis was associated with short-term pain reduction, its effect on motor recovery cannot be determined from the present follow-up period [23].

This aligns with the understanding that functional recovery in chronic neuropathies requires multimodal approaches beyond isolated surgical intervention. The short evaluation period may also have been insufficient to detect measurable motor improvement. Although not all patients demonstrated gains in motor function, stabilization of the disease without further symptom progression met patient expectations, likely due to clear preoperative counseling regarding the limitations of surgery, which contributed to better treatment adherence and overall satisfaction.

Our methodology demonstrated that the significant short-term impact of neurolysis was mainly limited to pain reduction at the group level across operated nerve groups, sustained throughout follow-up, including in 27.7% of chronic cases. This overall pain reduction represents a relevant finding, although individual responses were variable, and additional studies are needed to explore the mechanisms behind transient sensitivity improvement exclusively in common fibular nerve cases. Our findings extend previous evidence on decompressive surgery in leprosy neuritis by providing prospective, nerve-specific estimates of short-term sensory, motor, and pain outcomes. In particular, the robust and sustained analgesic effect across all nerve groups, contrasted with minimal sensory and motor recovery, supports the interpretation of neurolysis as a pain-relief procedure rather than as an intervention that reliably reverses established sensory or motor deficits in the short term. The immediate pain relief observed supports the hypothesis that mechanical decompression rapidly restores endoneurial microcirculation, alleviating ischemic pain independent of structural nerve repair [26].

The significant group-level reduction in pain observed after neurolysis may reflect the relief of mechanical and ischemic components of leprosy neuropathy. One plausible explanation is that decompression of enlarged nerve trunks at fixed anatomical tunnels reduces local pressure and improves intraneural perfusion, thereby alleviating an ischemic component of neuropathic pain. Release of constricting perineural tissues may also decrease traction and local irritation during limb movement, particularly in nerves that cross narrow osteofibrous canals. These mechanisms are biologically plausible and consistent with the pathology of leprosy neuritis and experimental models of nerve compression [4,5,26]. However, they cannot be directly demonstrated in the present cohort, because neurophysiological testing, imaging assessment, and intraoperative measures of nerve perfusion were not systematically performed. Therefore, the analgesic response observed in this study should be interpreted as a clinical association within the context of neurolysis and routine postoperative care, rather than as proof of a single pathophysiological mechanism [4,5].

Pain worsening in 7 patients from the < 3-year group also deserves consideration. Individual-level clinical information for these cases is provided in S1 Table. Re-review of these cases showed clinical heterogeneity: four patients had borderline leprosy, one tuberculoid, one lepromatous, and one pure neural leprosy; five underwent ulnar/median neurolysis and two common fibular/tibial neurolysis. Two patients had documented leprosy reactions before neurolysis, one type 1 and one type 2; one had diabetes; and one had severe chronic axonal ulnar neuropathy with active denervation on electromyography. Thus, no single mechanism can be inferred. Although neurolysis had an overall analgesic effect, early postoperative worsening may occur in some patients and may reflect transient local inflammation, surgical manipulation of a diseased nerve, active or subclinical neuritis, reactional activity, ongoing leprosy-related nerve injury, or advanced axonal damage. Persistent or worsening neuropathic pain may also occur when central sensitization or immune-mediated nerve injury predominates over mechanical compression. Clinically, this finding reinforces the need for careful postoperative monitoring, individualized pain management, and realistic preoperative counselling.

Taken together, these findings suggest that when sensory loss or paralysis reflects advanced structural nerve damage, neurolysis is unlikely to restore established deficits within the early postoperative period. In such cases, its main clinical value may lie in pain relief and, potentially, in preventing further deterioration when ongoing compression or inflammation is present.

This study has some limitations. Our follow-up window (30–60 days) captures early postoperative changes, but it is likely too short to fully characterize longer-term sensory and, especially, motor recovery after decompression; delayed improvement may occur in some patients depending on baseline axonal damage and the time course of remyelination and reinnervation. Likewise, although pain relief was robust in the early postoperative period, its durability beyond 60 days cannot be determined from these data. The absence of a non-surgical comparison group restricts causal attribution of the observed changes exclusively to neurolysis and makes it difficult to disentangle surgical effects from the natural course of leprosy neuritis and reactional states. Leprosy reactions should also be considered when interpreting the outcomes. Reactional episodes may independently increase nerve inflammation, pain, sensory impairment, and motor dysfunction, while their treatment, particularly systemic corticosteroids and thalidomide, may modify clinical trajectories. Although we summarized the frequency, type, timing, and management of documented reactions by clinical form, reaction severity, exact duration, and treatment course were not standardized by the study protocol. Therefore, residual confounding related to reactional activity and anti-reactional therapy cannot be excluded. The interpretation of pain outcomes should also consider concomitant medication use. Analgesics, non-steroidal anti-inflammatory drugs, systemic corticosteroids, and medications for neuropathic pain were prescribed according to clinical need before, during, and after neurolysis, but were not assigned or standardized by the study protocol. Because dose, tapering schedule, and duration were not consistently available for all patients, these treatments may have contributed to pain reduction, particularly in the early postoperative period. Thus, the analgesic benefit observed in this cohort should be interpreted within the context of routine postoperative care, rather than as the isolated effect of surgical decompression, and residual confounding cannot be excluded. In addition, heterogeneity in nerve involvement, baseline severity, and chronicity of neuropathy may have introduced residual confounding despite statistical adjustment. Quality-of-life data were also not prospectively collected, which limited our ability to assess the broader patient-centered impact of neurolysis on daily activities, work capacity, and psychosocial well-being [27]. Future studies should incorporate validated quality-of-life instruments as clinically relevant endpoints. Finally, the lack of systematic neurophysiological testing and/or imaging assessments precluded objective correlation between clinical trajectories and structural or functional nerve measures over time. These factors should be considered when interpreting the findings and reinforce the need for longer-term controlled studies incorporating validated quality-of-life instruments to refine surgical indications and clarify the role of neurolysis in functional recovery.

## Conclusion

Among patients with leprosy neuropathy whose symptoms persisted despite clinical treatment, neurolysis, within the context of routine postoperative care, was associated with significant short-term reduction in neuropathic pain at the group level. However, this response was not universal, as 24.2% of patients did not experience pain reduction and a subset of patients in the < 3-year group reported worsening pain after surgery. In contrast, sensory recovery was modest and transient, and no measurable gain in motor strength was observed during the 60-day follow-up. These findings support a pragmatic interpretation of neurolysis in this setting: it may offer meaningful pain relief, but should not be presented to patients as a procedure that reliably restores established sensory or motor deficits. Careful timing, patient selection, and realistic preoperative counselling remain essential, and longer-term controlled studies are needed to define more precisely its role in functional recovery.

## Supporting information

S1 Table De-identified supplementary clinical data. The table includes sex, leprosy classification, operated nerve(s), enlarged nerves, pain on nerve palpation, medication use, electromyography findings, diabetes diagnosis, leprosy reactions, and postoperative pain-worsening data for the 65 participants included in the study. “Pain worsening after neurolysis” identifies participants who reported increased pain after surgery during follow-up. Abbreviations: EMG, electromyography; MDT, multidrug therapy for leprosy.
(XLSX)
